# Gender Differences in Empathic Sadness towards Persons of the Same- versus Other-sex during Adolescence

**DOI:** 10.1007/s11199-016-0649-3

**Published:** 2016-06-27

**Authors:** Suzannah Stuijfzand, Minet De Wied, Maaike Kempes, Jolien Van de Graaff, Susan Branje, Wim Meeus

**Affiliations:** 1 Research Centre Adolescent Development, Utrecht University, Martinus J. Langeveldgebouw, Heidelberglaan 1, 3584CS Utrecht, The Netherlands; 2 Netherlands Institute for Forensic Psychiatry and Psychology, Gansstraat 170, 3582EP Utrecht, The Netherlands; 3 Tilburg School of Social and Behavourial Sciences, Tilburg Univeristy, Warandelaan 2, 5037 AB Tilburg, The Netherlands

**Keywords:** Same-sex versus other-sex affective empathy, Gender differences, Adolescence, Empathy development, Affective empathy

## Abstract

Although gender differences in affective empathy are well established, evidence of gender differences in the development of affective empathy is inconsistent. Consideration of same-sex versus other-sex affective empathy may assist in elucidating these inconsistencies. Gender differences were investigated in the experience of empathic sadness towards same- versus other-sex targets. The relationships were studied cross-sectionally (*N* = 730) and longitudinally (*N* = 318) with Dutch adolescents using the empathic sadness scale of the Index of Empathy for Children and Adolescents (IECA; Bryant 1982). In both studies, female adolescents reported more empathic sadness than did male adolescents. Female targets also received more affective empathy than did male targets, and, more importantly, gender differences were observed in same-sex versus other-sex affective empathy. Specifically, in both studies male adolescents reported less empathic sadness towards same-sex than towards other-sex targets. In contrast, female adolescents reported more empathic sadness towards same-sex than towards other-sex targets in the cross-sectional study, and equal levels of empathic sadness towards both types of targets in the longitudinal study. Findings highlight the importance of considering same-sex versus other-sex affective empathy. Gender differences in same-sex and other-sex affective empathy have implications for assisting adolescents in social conflict resolution and interventions for bullying and aggressive behaviour in adolescence using empathy training.

Empathy, the vicarious experience of another’s feelings, is important for moral understanding and motivating prosocial behaviour (Eisenberg et al. 2005; Hoffman 2008). Empathy also plays a pivotal role in inhibiting unwanted adolescent behaviours such as delinquency and aggression (Jolliffe and Farrington 2004). As such, empathy is important for maintaining functionality on an individual and societal level. Females report higher empathic responding than do males, and this gap between genders increases during the transition to adolescence (Eisenberg and Lennon 1983; Mestre et al. 2009; Van der Graaff et al. 2014). Differential gender socialisation theories and consideration of social and biological changes occurring at adolescence point to gender differences in affective empathy development during adolescence (Oransky and Marecek 2009). However, empirical evidence for this prediction is inconsistent. Consideration of empathic responses to same-sex and other-sex others would assist in ironing out these inconsistencies. Gender differences in same-sex versus other-sex affective empathy, specifically in terms of empathic sadness, have been established in three cross-sectional studies conducted in the United States (Bryant 1982; Feshbach and Roe 1968) and Norway (Olweus and Endresen 1998). The current study attempts to replicate these findings in a sample of Dutch adolescents, as well as expand on them by adding a longitudinal study allowing investigation of gender differences in the *development* of adolescents’ same-sex versus other-sex empathy.

## Affective Empathy and Gender Differences

Empathy has been defined in terms of cognitive and affective empathy (Eisenberg and Lennon 1983; Hoffman 2008). *Cognitive empathy* refers to understanding another person’s feelings, that is, taking their perspective. *Affective empathy* is the ability to share emotions. Within affective empathy there can be an emotional matching of another’s response (pure empathy), feeling *for* another person (sympathy/compassion) or feeling distress in response to another’s distress (personal distress) (Gruen and Mendelsohn 1986). Indices of empathy, such as the one used in the present study, often involve the assessment of emotional matching, as well as sympathetic responding and personal distress reactions (Miller and Eisenberg 1988). Furthermore, affective empathy can be measured as either dispositional or state empathy. *State empathy* is defined as empathic responsiveness to a given situation and is thought to be transient over contexts and situations (De Wied et al. 2005). *Dispositional or trait empathy*, rather, refers to the general ability to share the observed emotions of others and is reminiscent of a personality trait (De Wied et al. 2007a)—for example, sharing another’s sadness, anger or pain. The current study focuses on dispositional affective empathy, specifically empathic sadness.

Gender differences in affective empathy, with females showing higher affective empathy than do males, are well established in the literature across ages (Eisenberg and Lennon 1983; Knafo et al. 2008; Lam et al. 2012), and this gap increases in adolescence (Lam et al. 2012; Van der Graaff et al. 2014). Females’ empathic behaviours may have a more affective basis than males’ (Derntl et al. 2010) prior to adolescence through early socialisation (Hoffman 1977). Such differences may further increase in adolescence given the biological and social changes that occur within this period, which have the potential to facilitate or inhibit associations with affective empathy.

During adolescence both genders show an increase in sex hormones (Buchanan et al. 1992). Male adolescents show an increase in testosterone, which has been causally linked to depletion in empathy (Hermans et al. 2006), whereas female adolescents show an increase in oestrogen which could be plausibly linked to promotion of empathy through its impact on empathic facilitator oxytocin (Buchanan et al. 1992; Decety 2011; cf. Yildirim and Derksen 2012). This divergence suggests that potential gender differences in empathy develop over adolescence as puberty progresses, with an increase in empathy being more likely in female adolescents.

Differential gender socialisation theories also suggest the presence of gender differences in affective empathy development over adolescence, particularly as the adolescent peer context pressures individuals to conform to gender-specific behaviour (Oransky and Marecek 2009; Pettitt 2004). It has been suggested that changes occurring within the adolescents’ social world, such as new friendships from moving schools, may influence social cognition (Blakemore and Choudhury 2006), of which empathy is part. This social change occurs as the physical changes that occur in puberty signal the move from childhood to adulthood and adult gender roles, which leads peer-socialisers to exert pressure on the group to behave in a gender-typed way (Pettitt 2004; Rose and Rudolph 2006). The female adolescent’s social world appears to encourage empathy where young women spend more time talking over problems together and show more pro-social behaviour to each other than males show to each other, necessitating the use of empathy in female interactions. Adolescent males care more about social dominance and competitive sporting activities (Rose and Rudolph 2006); such behaviours do not facilitate empathic behaviours.

These gender differences in same-sex peer relationships become more consistent with age (Rose and Rudolph 2006). Between the ages of 9 to 16 years-old, on average, male adolescents show higher impassivity—indicating that males tend not to express emotions, showing lower sensitivity and loyalty than do female adolescents (Tello et al. 2012), which is not surprising given that these are considered feminine characteristics (Pettitt 2004). This trend may have implications for adolescents’ affective empathic behaviour. Irrespective of biological gender, femininity facilitates affective empathic tendencies, whereas masculinity inhibits these same tendencies (Karniol et al. 1998).

Despite these theoretical suggestions, support for gender-specific changes in adolescent affective empathy is inconsistent. Cross-sectional studies have found female adolescents’ affective empathy to be higher in late adolescence compared to early and middle adolescence, whereas affective empathy in male adolescents did not differ by age (Olweus and Endresen 1998; Van Tilburg et al. 2002). In addition, in these studies female adolescents consistently showed higher empathy than did male adolescents across all ages. One further cross-sectional study found that female adolescents showed higher levels of affective empathy than did male adolescents, but it found no age differences (Adams et al. 1979).

Longitudinal studies consistently found that female adolescents showed higher levels of affective empathy over time. Some studies demonstrated an increase in affective empathy for both genders where gender differences either remained stable over time (Davis and Franzoi 1991) or increased (Mestre et al. 2009). However, other studies showed stable levels of affective empathy from age 13 to 18 years-old for female adolescents, but a temporary decrease followed by an increase from middle adolescence for male adolescents (Van der Graaff et al. 2014), or stable levels of affective empathy over time for both genders (Eisenberg et al. 2005). The use of different measures (e.g., the Interpersonal Reactivity Index by Davis 1980; the IECA by Bryant 1982; the Mehrabian and Epstein 1972, Empathy Scale) and designs (cross-sectional or longitudinal) may be interfering with the consistency of results, as might the lack of consideration of same-sex versus other-sex affective empathy.

## Same-Sex versus Other-Sex Affective Empathy

Feshbach and Roe (1968) were the first known to investigate same-sex versus other-sex affective empathy in children**.** They found that 6–7 year-old children showed more empathy towards a child of the same-sex than of the other-sex. Bryant (1982) also demonstrated that during childhood affective empathy, specifically empathic sadness for same-sex others, was stronger than for other-sex others, but found that this pattern changed in adolescence, particularly for male adolescents. Whereas girls show more empathic sadness towards their own sex at all ages, boys show increasingly more empathic sadness towards the other-sex at later ages, 12–13 years-old. These results on empathic sadness were confirmed in an adolescent cohort (Olweus and Endresen 1998). Overall, female adolescents showed more empathic sadness towards both genders than did males regardless of age. For male adolescents, same-sex empathic sadness decreased with age whereas other-sex empathic sadness increased.

An evolutionary perspective, based on principles of sexual selection and male competition, may explain the pattern in empathy for male adolescents (Olweus and Endresen 1998). On one hand, the growing interest in and attraction to the other-sex in adolescence may enhance male adolescents’ empathic responses towards female adolescents (Tello et al. 2012), particularly given that co-operation and feelings of nurturance promote empathy (Batson et al. 2005; Lanzetta and Englis 1989). On the other hand, the (entry to a) masculine competitive environment may inhibit empathic responding to male competitors (Tello et al. 2012). Indeed, competition is associated with the depletion of empathy (Lanzetta and Englis 1989). During adolescence, competition among males may overrule the influence of characteristics such as similarity and familiarity (Ma et al. 2011), which normally promote empathy (Davis 1994; Preston and De Waal 2002). Competition has also been linked to increases in testosterone, which occurs in adolescence (Buchanan et al. 1992; Mazur 1985), and testosterone itself is causally linked to empathy inhibition (Hermans et al. 2006). However, competition is not a general feature of same-sex friendships in female adolescents (Hartup 1992). Competition and sexual activities become most salient in adolescence (Gallup et al. 2010), and male adolescents’ and female adolescents’ differential responses to these adolescent contextual characteristics make it plausible that gender differences in same-sex versus other-sex empathy could be expected.

## The Present Study

Gender differences in same-sex versus other-sex affective empathy, specifically empathic sadness, will be examined cross-sectionally (Study 1) in a sample of adolescents. Although cross-sectional studies may suggest patterns of development, only longitudinal studies explicitly investigate development over time. Therefore, these patterns will then be investigated longitudinally (Study 2) allowing developmental influences on gender differences in same-sex versus other-sex empathy to be assessed in a two-wave longitudinal sample of adolescents. Given theory and evidence regarding gender differences in adolescents’ affective empathy, as well as current theory concerning the influence of competition and sexual activities on affective empathy, we propose three hypotheses. First, female adolescents are hypothesised to score higher than male adolescents on empathic sadness overall (Hypothesis 1). Second, as per previous research, we expect increasing scores of empathic sadness over grades/time, especially for female adolescents (Hypothesis 2). Given the inconsistencies in previous research regarding affective empathy, in male adolescents, there are no specific expectations for male adolescents across grades/over time. Third, gender differences are expected in same-sex and other-sex empathy, with female adolescents showing more same-sex than other-sex empathic sadness, but male adolescents showing the reversed pattern (Hypothesis 3).

## Study 1: the Cross-Sectional Study

### Method

All procedures performed in studies involving human participants were in accordance with the ethical standards of the institutional and/or national research committee and with the 1964 Helsinki declaration and its later amendments or comparable ethical standards. Informed consent was obtained from all individual participants included in our study.

#### Participants

The adolescents from Study 1 took part in a larger study investigating the effects of the Stay in Love + program developed to diminish dating violence among adolescents (Kempes et al. 2010). The original sample consisted of 876 adolescents, of which 338 attended grade seven, 362 attended grade eight, and 176 attended grade nine. The schools were selected to reflect the degree of urbanization in the Netherlands, that is, five schools were situated in cities and five schools in towns, and degree of religious denomination, that is, catholic, protestant, or public. The majority (*n* = 755) of the adolescents attended lower vocational secondary education; 121 adolescents attended higher vocational secondary or pre-university education. However, because the original study’s design was catered to the lower vocational stream, and participants in the higher education were only from the first grade (thus, they were not equal distributed across grades), those with higher education were omitted to ensure a homogeneous sample. In total, 25 adolescents were not present at the first measurement, when affective empathy was measured.

Therefore, the usable sample of the current study consisted of 730 adolescents, with 310 female adolescents (43 %). Age was available for 722 participants. There were no age differences between female (*M* = 14.09, *SD* = 1.03) and male (*M* = 14.01, *SD* = 1.03) adolescents, *t*(720) = −1.02, *p* = .307. Age ranged from 12 to 15 years-old in grade seven, 13–16 years-old in grade eight, and 14–17 years-old in grade nine. There was a significant difference in the distribution of females and males within school grade, but with a small effect size, χ^2^(2) = 9.66, *p* = .008, *φ* = .12. There were more females than males in grade eight (188 female adolescents, 119 male adolescents) and in grade nine (84 female adolescents, 47 male adolescents). Ethnicity was available for 727 participants. The majority of participants were Dutch (*n* = 490); the remainder (*n* = 237) belonged to an ethnic minority group, having at least one parent born in another country (unspecified). Ethnic minority groups in the Netherlands include Moroccan, Turkish, and Surinamese. Which groups are represented within the ethnic minority group here is unknown.

#### Measures

A Dutch version of the self-report 22-item Index of Empathy for Children and Adolescents (IECA, Bryant 1982) was used to measure adolescent dispositional affective empathy. The IECA is believed to be the only questionnaire measure allowing the investigation of same-sex versus other-sex affective empathy and was used by Bryant (1982) and by Olweus and Endresen (1998). The IECA has shown good construct validity and convergent validity (Bryant 1982). De Wied et al. (2007b) investigated the structure of the Dutch IECA and identified two factors: an empathic sadness factor, with good reliability and construct validity, and an attitude factor, with weak reliability. Based on these findings, Study 1 (and Study 2) used the empathic sadness factor. This factor contains one item specifically targeting one’s affective empathic response to sadness: “Some songs make me so sad I feel like crying” and three pairs of mirror items. These mirror items have identical wordings except for the sex of target (boy or girl): “I get upset when I see a girl [boy] being hurt,” “It makes me sad to see a girl [boy] who can’t find anyone to play with,” and “Seeing a girl [boy] who is crying makes me feel like crying.” One item from each pair was used to create two subscales reflecting affective empathy towards females and affective empathy towards males.

The IECA can be used in different formats with either a binary, 6- or 9-point rating scale, and higher scores indicate higher affective empathy. In Study 1, the IECA was answered on a binary yes (coded 1)/no (coded 0) scale. Three scores were generated from the IECA. First, the seven items of the empathic sadness factor were summed to create an overall empathic sadness score (min: 0, max: 7). The target/gender-appropriate items from each of the three pairs of mirror items were summed (min: 0, max: 3) to create two subscales: affective empathy toward male targets and toward female targets.

#### Procedure

Data collection in the original study consisted of four measurement occasions in a classroom setting during school hours spaced 1 month apart and was approved by the Ethics Board of the Faculty of Social Sciences of Utrecht University. Only the first measurement occasion contained questions about gender, age, ethnicity, and empathy. Before the first measurement occasion, the schools informed parents about the purpose of the study, and parents were able to refuse consent for their child’s participation by returning a written form. None of the parents refused consent. Before the start of the first measurement, all students were informed about the purpose of the research and the requirements of participation; confidentiality of their responses was assured. After brief verbal instructions, questions were presented on a laptop and read out loud through headphones connected to the laptops. Items were presented on a computer screen where one item had to be completed before the next item was displayed ensuring completion of all questionnaire items. Students were encouraged to ask the supervisor for help when needed. Small rewards (sweets) were provided after completion of all questionnaires.

#### Strategy of Analysis

Prior to analysis, the presence of outliers was checked and assumptions of normality and sphericity were checked and met. Interpretations of effect sizes were based on Cohen (1988), and post hoc tests were corrected for alpha inflation using Tukey’s HSD. All analyses were conducted in SPSS20. An ANOVA was conducted on the empathic sadness scale to test Hypotheses 1 and 2, with between factors gender of respondent and school grade. School grade, rather than age, was used as the between factor in Study 1 for several reasons. First, use of grade enabled replication of the previous adolescent study (Olweus and Endresen 1998). Second, age was not available for all participants, whereas grade was, therefore use of grade maximised power. Third, grade also involves age and education level as well as acknowledges the social interactions between different ages within grades (i.e. grade seven age range was 12–15 years-old within one variable). Finally, splitting the sample by age would have resulted in very small groups for the youngest and oldest children, with much larger groups for the ages in between and thus preventing accurate group comparisons. Splitting children by grade resulted in equal groups. Nevertheless, when the same analyses were conducted with age as the between factor rather than grade, the overall results were the same as reported here.

To test Hypothesis 3, a repeated measures ANOVA was conducted. The within factor was gender of target (“girl” or “boy” items), with between factors of gender of respondent and school grade. Correlations between the affective empathy subscales, reflecting affective empathy towards females and males, showed a positive strong relationship for the total sample (*r* = .77, *p* < .001), female targets (*r* = .74, *p* < .001), and male targets (*r* = .72, *p* < .001). Given these high correlations and the identical wording of the items making up the subscales (only gender of target differs), we can assume that the subscales measure the same construct, allowing for the use of the repeated measures ANOVA.

### Results

#### Gender Differences in Adolescents’ Empathic Sadness

The significant main effect of gender of respondent, *F*(1, 724) = 180.17, *p* < .001, ηp^2^ = .20, with a large effect size, indicates that female adolescents report higher empathy than did male adolescents (∆*M* = 2.00). There was no main effect of grade, *F*(2, 724) = .29, *p* = .750. Therefore, overall there was no linear increase or decrease in empathy across grades. There was no significant interaction between gender of respondent and grade, *F*(2, 724) = 2.22, *p* = .110. Support was therefore found for Hypothesis 1 which predicted that female adolescents would score higher on overall empathic sadness than would male adolescents, but not for Hypothesis 2 which predicted increasing empathic sadness scores over grade/time, particularly for girls.

#### Gender Differences in Adolescents’ Same-Sex versus Other-Sex Empathic Sadness

Results demonstrate a significant main effect of gender of respondent, *F*(1, 724) = 115.39, *p* < .001, ηp^2^ = .14) with a large effect size, where on average, female adolescents show higher empathy than did male adolescents (∆*M* = .74, *p* < .001). There was a significant main effect of gender of target, *F*(1, 724) = 58.01, *p* < .001, ηp^2^ = .07, with a medium effect size, where empathy towards female targets was on average higher than was empathy towards male targets (∆*M* = .20). There was no main effect of grade, *F*(2, 724) = .14, *p* = .870, indicating there was no overall linear increase or decrease in empathy across grades. There were no significant two-way interactions between the variables (*F*s = 1.18–1.66, *p*s > .100).

**Fig. 1:**
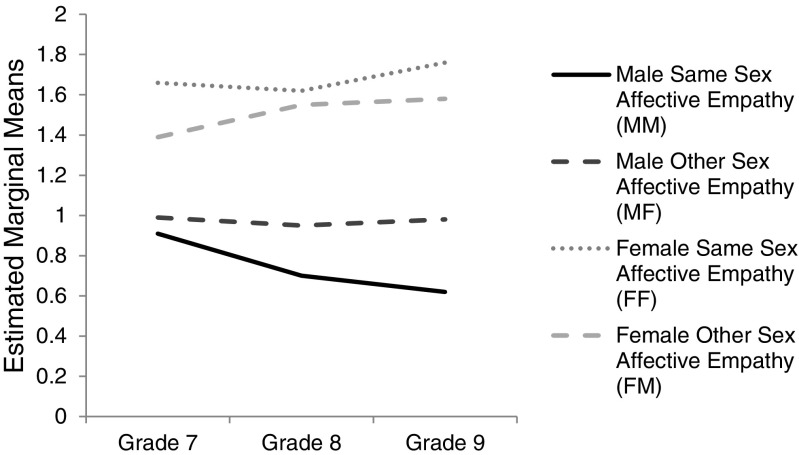
*Line graph* to illustrate average Affective Empathy score (scale range 0 to 2) as a function of same-sex versus other-sex affective empathy, gender, and grade in cross-sectional data (Study 1). *N* = 730

However, there was a significant three-way interaction between gender of target, gender of respondent, and grade, *F*(2, 724) = 6.76, *p* = .001, ηp^2^ = .02, with a small effect size. This interaction is illustrated in Fig. 1. To further investigate this three-way interaction, the analysis was repeated separately for male adolescents and for female adolescents. Once again there was a significant main effect of gender of target for female adolescents, *F*(1, 417) = 24.72, *p* < .001, ηp^2^ = .06, and for male adolescents, *F*(1, 307) = 35.65, *p* < .001, ηp^2^ = .10, with medium effect sizes. As before, there was no significant main effect of grade for female adolescents, *F*(1, 417) = .73, *p* = .485, or for male adolescents, *F*(1, 307) = 1.22, *p* = .295. However, there was a significant interaction between gender of target and grade for female adolescents, *F*(1, 417) = 3.46, *p* = .032, ηp^2^ = .02, and for male adolescents, *F*(1, 307) = 4.68, *p* = .010, ηp^2^ = .03, with small effect sizes.

Follow-up paired samples *t*-tests, tested separately for female adolescents and male adolescents, were conducted to elucidate this interaction. For female adolescents, paired sample *t*-tests showed that empathy towards female targets was significantly different from empathy towards male targets in grade seven, with a large effect size, *t*(147) = 5.18, *p* < .001, *d* = .85, and in grade nine, with a medium effect size, *t*(83) = 2.35, *p* = .020, *d* = .52, with higher empathy towards female targets than towards male targets (grade seven: ∆*M* = .27; grade nine: ∆*M* = .18). There was no significant difference in grade eight, (*t*(187) = 1.45, *p* = .149.

A different pattern was revealed for male adolescents. Paired sample *t*-tests showed that empathy towards female targets was significantly different from empathy towards male targets in grade eight, with a medium effect size, *t*(118) = 4.13, *p* < .001, *d* = .76, and in grade nine, with a large effect size, *t*(46) = 4.10, *p* < .001, *d* = 1.21. Empathy towards female targets was, on average, higher than empathy towards male targets (grade eight: ∆*M* = .25; grade nine: ∆*M* = .36). There was no significant difference in grade seven, *t*(143) = 1.78, *p* = .077. An ANOVA revealed a significant difference in same-sex empathy in male adolescents between grades 7 and 9, *F*(2, 307) = 3.04, *p* = .049, ηp^2^ = .02, with a small effect size. Inspection of the means suggests same-sex empathy in male adolescents decreases as grade increases. Thus support was found for Hypothesis 3 which predicted that female adolescents would show more empathic sadness towards targets of the same-sex than towards targets of the other-sex, whereas males would show the reverse pattern.

### Discussion

Gender differences in empathic sadness were found in the cross-sectional study. First, as hypothesised, female adolescents showed higher empathic sadness than did male adolescents. Interestingly, not only did female adolescents reported more empathy, but female targets also *received* more empathy than did male targets, as demonstrated by male and female adolescent reported empathy towards female targets. Second, different from expectations, we found no overall association between grade and empathy. Third, in line with hypotheses, the three-way interaction indicated that female and male adolescents did not show the same pattern of same-sex and other-sex empathy across grades. Female adolescents showed higher same-sex empathy, whereas male adolescents showed higher other-sex empathy. This pattern of results, established within a Dutch dataset, replicate and support the findings of Norwegian (Olweus and Endresen 1998) and U.S. (Bryant 1982) studies. Although cross sectional studies may suggest patterns of development, only longitudinal studies explicitly investigate development over time. Therefore, these patterns were investigated longitudinally (Study 2) allowing developmental influences on gender differences in same-sex versus cross-sex empathy to be assessed.

## Study 2: the Longitudinal Study

### Method

#### Participants

Participants were from the ongoing longitudinal study CONflict And Management Of RElationships (CONAMORE). Empathy was assessed in the second and third wave (1 year apart) of the family sample, being a specific subsample of the complete CONAMORE sample consisting of 323, two-parent Dutch families. There was no attrition between the second and third waves. (See Van Doorn et al. 2011, for a full description of the sample and procedure.) All but five participants (2 female adolescents and 3 male adolescents) completed the questionnaires at both time points.

Once incomplete cases were removed, 318 cases (164 female adolescents, 55 %) were included in our final analysis. Almost all participants (99 %) were Dutch. The remaining 1 % classified themselves as “Other” (choices being: Dutch, Surinamese, Moroccan, Turkish or Other). At the second wave (Time 1), age ranged from 13 to 16 years-old (*M* = 14.41, *SD* = .55). All participants were in Dutch Secondary School education: approximately 49 % at schools preparing for university, 34 % preparing for higher education, and 17 % for lower- level jobs. The majority of participants were in grade nine (98 %). There was no significant difference in the distribution of gender between education levels, χ^2^(7) = 6.67, *p* = .464, or grade, χ^2^(1) = .19, *p* = .664).

#### Measures

Description and psychometric properties of the three subscales of the IECA are reported in Study 1. Adolescents answered the IECA (Bryant 1982) on a 9-point Likert scale from −4 (*strongly disagree*) to +4 (*strongly agree*) at two time points. The seven items of the overall empathic sadness factor were averaged to create the empathic sadness score at both time points (min: −4, max: +4), which showed good internal consistency reliability (Time 1 *α* = .86; Time 2 *α* = .86). The three pairs of mirror items were averaged to create two subscales (min: −4, max: 4), representing affective empathy towards female targets and towards male targets and showing acceptable internal consistency reliability at Time 1 (female adolescents, *α* = .65; male adolescents, *α* = .70) and Time 2 (female adolescents, *α* = .69; male adolescents, *α* = .71). All scales were normally distributed.

#### Missing Items

After incomplete cases were removed, eight participants showed missing item scores on the IECA. All eight participants missed different items; therefore missingness was not related to the content of a particular item. Relative means substitution was used to impute data. This method is appropriate when one or two items are missing per case and reflects the ranking of the participant in the sample correcting for items having different means (Raaijmakers 1999).

#### Procedure

Families invited to take part were given written information regarding the study’s requirements and written consent was required from adolescents and parents prior to participation. Adolescent participants answered paper-and-pencil questionnaires within school, as well as additional questionnaires at home. Families (3 participants) were compensated with €27, with an addition €10 for school participation, per wave.

#### Strategy of Analysis

Prior to analysis the presence of outliers was checked, and assumptions of normality and sphericity were checked and met. Interpretations of effect sizes were based on Cohen (1988), and post hoc tests were corrected for alpha inflation using Tukey’s HSD. All analyses were conducted in SPSS20. Test-retest Pearson’s correlations were computed to check that the empathy subscales measured the same construct over time. Also, test-retest correlations provide an indication of the continuity of constructs over time (Roberts et al. 2001). To test Hypothesis 1 and 2, a repeated measures ANOVA was conducted with empathic sadness at Time 1 and Time 2 as the within factor and gender of respondent as the between factor. As in Study 1, correlations between the empathy subscales were strong and positive, at each time point, within the total sample, for female targets, and for male targets (see Table 1), suggesting that the two subscales measured the same construct. Therefore, to test Hypotheses 3 a multivariate repeated measures ANOVA was conducted wherein the two within factors were time and gender of target and gender of respondent was the between factor.

**Table 1:** Correlations among affective empathy measures for males, females, and the total sample within the CONAMORE longitudinal sample, study 2

Affective empathy measures	1	2	3
Females (*n* = 165)			
1. Time 1: Affective empathy towards females	--		
2. Time 1: Affective empathy towards males	.67**	--	
3. Time 2: Affective empathy towards females	.78**	.53**	--
4. Time 2: Affective empathy towards males	.53**	.51**	.82**
Males (*n* = 157)			
1. Time 1: Affective empathy towards females	--		
2. Time 1: Affective empathy towards males	.67**	--	
3. Time 2: Affective empathy towards females	.71**	.54**	--
4. Time 2: Affective empathy towards males	.62**	.62**	.78**
Total sample (*N* = 318)			
1. Time 1: Affective empathy towards females	--		
2. Time 1: Affective empathy towards males	.74**	--	
3. Time 2: Affective empathy towards females	.79**	.64**	--
4. Time 2: Affective empathy towards males	.71**	.68**	.85**

*p < .01. ** *p* < .001

### Results

Test-retest Pearson’s correlations between affective empathy towards female and towards male targets at Time 1 and Time 2 were significant and positive, and they showed large effect sizes within the total sample as well as for female and for male targets (see Table 1). Thus, the constructs of affective empathy towards females and towards males showed relative stability over time.

#### Gender Differences in Adolescents’ Empathic Sadness

When investigating gender differences longitudinally using all seven items, we found a significant main effect of gender of respondent, *F*(1, 316) = 567.59, *p* < .001, ηp^2^ = .37, with a large effect size. Female adolescents on average showed higher empathy than did male adolescents (∆*M* = 1.89, *p* < .001). There was also a main effect of time, *F*(1, 316) = 6.73, *p* = .010, ηp^2^ = .02, with a small effect size. Empathic sadness was, on average, higher at Time 2 than at Time 1(∆*M* = −.16), indicating an overall increase in empathy over time. There was no significant interaction between time and gender of respondent, *F*(1, 316) = .03, *p* = .855, thus change over time was equal between genders. Support was therefore found for Hypothesis 1 which predicted that female adolescents would score higher on overall empathic sadness than would male adolescents, and for Hypothesis 2 which predicted increasing empathic sadness scores over grade/time, particularly for girls.

#### Gender Differences in Adolescents’ Same-Sex versus Other-Sex Empathic Sadness

In the longitudinal investigation of gender differences within the subscales, a significant main effect of gender of respondent, *F*(1, 316) = 132.61, *p* < .001, ηp^2^ = .30, with a large effect size, was found, indicating that female adolescents showed on average higher empathy than did male adolescents (∆*M* = 1.67). There was a main effect of time, *F*(1, 316) = 4.92, *p* = .027, ηp^2^ = .02, with a small effect size. Empathy at Time 2 was on average higher than at Time 1 (∆*M* = .13). This difference indicates that there was an overall linear increase in empathy over time. Also the significant main effect of gender of target, *F*(1, 316) = 7.34, *p* = 007, ηp^2^ = .02, with a small effect size, shows empathy towards female targets was on average higher than towards male targets (∆*M* = .14).

**Fig. 2:**
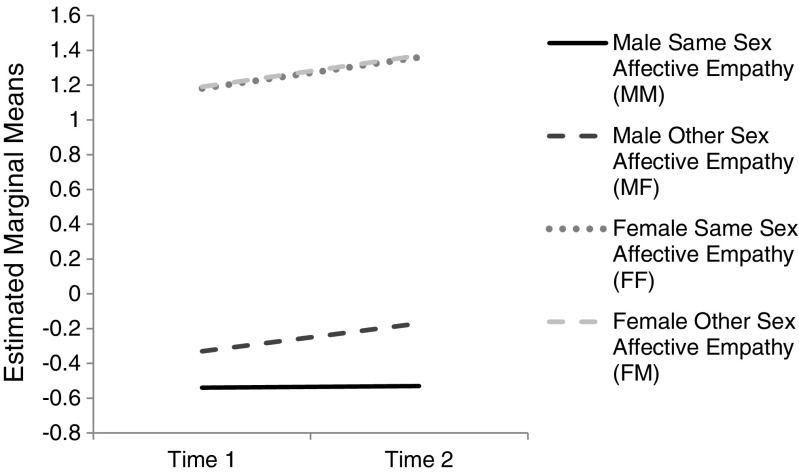
*Line graph* to illustrate Average Affective Empathy score (scale range − 4 to +4) as a function of same-sex versus other-sex affective empathy, gender, and time in Study 2. *N* = 318

There was no significant interaction between time and gender of respondent, *F*(1, 316) = .67, *p* = .413, indicating that change over time was equal between genders. Nor was there a significant interaction between gender of target and time, *F*(1, 316) = 1.21, *p* = .273. However, there was a significant interaction of gender of respondent and gender of target, *F*(1, 316) = 8.81, *p* = .003, ηp^2^ = .03, with a small effect size. Patterns of same-sex versus other-sex empathy are different for female adolescents and male adolescents (see Fig. 2). There was no significant interaction among gender of target, time, and gender of respondent, *F*(1, 316) = 1.25, *p* = .264.

To further investigate the significant two-way interaction between gender of target and gender of respondent, further analyses were conducted. Paired sample *t*-tests analysed the difference between empathy towards female targets and towards male targets at Time 1 and Time 2, separately for both genders. No significant differences were found for female adolescents at Time 1, *t*(163) = −.14, *p* = .892, or at Time 2, *t*(163) = −.21, *p* = .834. This further confirms that same-sex and other-sex empathy are similar over time for female adolescents. For male adolescents, there was no significant difference at Time 1, *t*(153) = 1.85, *p* = .066. However, there was a significant difference at Time 2, with a medium effect size, *t*(153) = 4.44, *p* < .001, *d* = .72, where higher empathy was reported towards female targets than towards male targets (∆*M* = .36). Thus partial support was found for Hypothesis 3 which predicted that female adolescents would show more empathic sadness to same-sex than to other-sex targets, whereas male adolescents would show the reverse pattern. Results indicated that female adolescents showed no difference in same-sex or other-sex empathic sadness, but male adolescent did show a different pattern. In line with Hypothesis 2 male adolescents showed more empathic sadness towards other-sex targets than towards same-sex targets.

### Discussion

Gender differences in empathic sadness were also found in the longitudinal study. First, as hypothesised, female adolescents showed higher overall empathy than did male adolescents. Interestingly, as in Study 1, female targets also received more empathy. Second, overall empathy was shown to increase over time for both genders. Third, results reveal gender differences in same-sex and other-sex empathic sadness, with female adolescents showing equal levels of same-sex and other-sex empathy, but male adolescents showing lower levels of same-sex than other-sex empathy. Study 2 confirms the results of Bryant (1982) and Olweus and Endresen (1998) and shows that gender differences in same-sex and other-sex empathy are not only present, but also stable over time.

## General Discussion

The goal of our research was to investigate whether there are gender differences in same-sex versus other-sex affective empathy. This was achieved by examining patterns cross-sectionally (Study 1) and longitudinally (Study 2). Hypothesis 1, that female adolescents would show higher levels of empathy than would male adolescents, was supported by both studies. Hypothesis 2, that female adolescents would show increasing levels of empathy was supported by Study 2. The main effect of time in Study 2 suggests an increase in overall empathy for both genders. Moreover, both studies reveal gender differences in same-sex and other-sex empathy supporting Hypothesis 3. In Study 1, female adolescents showed more same-sex than other-sex empathy, whereas male adolescents showed the reversed pattern. In Study 2, female adolescents showed equal levels of same-sex and other-sex empathy, whereas male adolescents, as in Study 1, showed less same-sex than other-sex empathy.

In agreement with previous findings (Olweus and Endresen 1998; Van Tilburg et al. 2002), Study 1 showed that female adolescents on average report higher empathy than do male adolescents across ages. Study 2 demonstrated that these gender differences were stable over time in adolescence. Females are more likely to have an affective response to the recognition of affect in another (Hoffman 2008), experience guilt, and are socialised to be empathic in a more affective way than are males (Hoffman 1977; Mestre et al. 2009). This pattern is in line with females’ greater reliance on emotion regions of their brains than males’ while carrying out general empathy tasks, even when task performance between genders is similar (Derntl et al. 2010).

Also in agreement with previous findings (Bryant 1982; Olweus and Endresen 1998), both studies demonstrated that not only do female adolescents report more empathy, but also female targets received more empathy than do male targets, particularly from male adolescents. It could be argued that, given that female adolescents are more likely to express sadness than are male adolescents (e.g., cry; Van Tilburg et al. 2002), young women may appear more vulnerable and therefore evoke more nurturing feelings from others than do young men. Nurturance, as an impulse to care and protect another, in itself explained empathic behaviour towards strangers more so than did similarity (Batson et al. 2005).

The overall linear increase in affective empathy found in Study 2 is in line with the findings of Davis and Franzoi (1991), yet contradicts previous findings where gender differences were found to increase over time (Mestre et al. 2009). Theories of differential socialisation may suggest gender differences in affective empathy development, but this was not supported by Study 2. Given the suggestion that pubertal processes activate urges related to affective drives (Dahl 2004), the overall linear increase found here is theoretically plausible. However, it is acknowledged that pubertal processes are gender-specific in timing and nature (Tanner 1971), thus the inclusion of pubertal processes in an investigation of same-sex versus other-sex empathy may elucidate gender differences in affective empathy development not evident here. Indeed, pubertal status has already been found to relate to a male decrease in affective empathy (Van der Graaff et al. 2014). Testosterone has been causally linked to empathy’s depletion (Hermans et al. 2006), and oestrogen could be plausibly linked to promotion of empathy through its impact on empathic-facilitator oxytocin (Buchanan et al. 1992; Decety 2011; cf. Yildirim and Derksen 2012). Therefore, the influence of pubertal processes may be worth considering in future research because they may assist in explaining the development found here*.* The increase in empathy by male adolescents towards female targets as seen in the increase in other-sex empathy for male adolescents may be driving the lack of gender difference in empathy development here. Additionally, perhaps the age range in Study 1 and the two wave limit in Study 2 were too restricting for such differences to be detected.

The current study demonstrates gender differences in same-sex versus other-sex empathy, both cross-sectionally and longitudinally. This consistent finding highlights the importance in considering the same-sex versus other-sex distinction in empathy development. There may be several explanations for the gender differences found. Competition and a lack of co-operation between males may explain why same-sex empathic sadness in male adolescents was, on average, lower than other-sex empathic sadness in both studies. Competition becomes most salient in adolescence (Gallup et al. 2010), with male adolescents’ friendships generally becoming more competitive (Hartup 1992); thus, young men may show less empathy towards each other (Lanzetta and Englis 1989). In contrast, same-sex empathic sadness in female adolescents, on average, increased between grades seven and nine. Although female adolescents’ friendships can be competitive under certain situations (Cronin Weisfeld et al. 1982; Gallup et al. 2011), female adolescents’ same-sex friendships are suggested to be intimate, close, and co-operative (Keener et al. 2012). Such characteristics promote empathy (Batson et al. 2005; Lanzetta and Englis 1989). Future studies could investigate competition or co-operation among peers as predictors of gender differences in same-sex versus other-sex empathy as potential mechanisms behind gender differences in adolescents’ empathy.

Also male adolescents look to female adolescents as potential sexual partners, and vice-versa (Forbes and Dahl 2010). Consideration of these developments may further assist in explaining the gender differences we found. It would certainly be beneficial for adolescents to show empathy to the other sex to make use of its social bonding facility in starting romantic relationships. The peer context of early–middle childhood is dominated by same-sex relationships (Rose and Rudolph 2006). However, during adolescence their peer network involving those of the other-sex increases (Galambos et al. 2009; Underwood and Rosen 2009). Over time adolescents’ relationships increasingly reflect their sexual interests (Galambos et al. 2009), and as peer relationships and romantic relationships develop, so too may adolescents’ use of empathy within interactions. As adolescents spend increasing amounts of time with the other sex, they may adopt parts of the other sex’s interaction style (Rose and Rudolph 2006). Thus given relationships with female adolescents appear to necessitate more empathic attributes, male adolescents may take on these relationship characteristics as they spend more time interacting with females; thus males show more empathic sadness towards females. If females do take on male relationship attributes, this may explain why their empathic sadness towards male targets in Study 1 was lower than their empathic sadness towards female targets. This of course happens over time and as peer relationships and romantic relationships develop, as may adolescents’ use of empathy within interactions. Therefore the pattern of empathic sadness shown in our two studies may also reflect the changing nature of adolescents’ peer relationships from same-sex cliques to more mixed-sex cliques.

It should be noted that details of the results between the studies do differ. Study 1 shows a decrease for same-sex empathic sadness in male adolescents across grades, whereas Study 2 shows an increase in total empathic sadness for both genders over time. Although the same measure was used in both studies, the response format did differ. Study 2 used a Likert scale, whereas Study 1 used a binary scale. However, even when using two different response formats, an overall consensus was reached from both studies: gender differences were seen in same-sex and other-sex empathic sadness. It is this overall pattern that is important when considering the contribution of results to the literature on empathy.

The difference in results between studies could, however, be due to individual differences within and between our samples, particularly given the general consensus that empathy is an individual difference factor (Jolliffe and Farrington 2004). This individual information is lost when comparisons are made at the mean level, as we did here. Also, given that Study 2 follows development, the distribution of individual differences maintains its source over time; however, this cannot be stated for Study 1. Including potential sources of individual differences when investigating same-sex versus other-sex adolescent empathy may assist in obtaining consistent results.

### Limitations and Future Research Directions

The current research inevitably has some limitations. First, it should be noted that results rely solely on a self-report questionnaire. This creates two issues, one being that we cannot account for how much shared source and method variance using this questionnaire over two time points has influenced the results. Future studies would benefit from multiple measures of affective empathy to ensure the longitudinal effects found here are robust.

Second, we cannot account for the influence of self-report biases due to gender-typed responding or social desirability (Zhou et al. 2003). Female adolescents may be more comfortable to report affective (same-sex and other-sex) empathy than are male adolescents given the gender differences encouraged by socialisation and the social context of adolescence (Eisenberg and Lennon 1983; Olweus and Endresen 1998). Indeed, objective measures of empathy utilising facial electromyography (fEMG) have found weaker gender differences in adolescents than those captured by self-report measures (Van der Graaff et al. 2016). However, these measures also may not be free of social desirability influences (Zhou et al. 2003). Also gender-specific characteristics become less important with age (Karniol et al. 1998), but considering our narrow age range, this demand characteristic may not be such an important influence. Considering the differences and changes occurring within adolescent social contexts (e.g., increasing competition between male adolescents), it may be that the items assessing empathy are not necessarily representative of how empathy is expressed between male adolescents. Future studies could explore this possibility by investigating whether how adolescent males express their empathy changes during adolescence, or whether male adolescents experience sympathy rather than empathic sadness in this context. Although the limitations of a single self-report measure are acknowledged and demand characteristics cannot be ruled out, they still only provides a limited explanation for the robust gender patterns demonstrated (Olweus and Endresen 1998).

Third, our study, as did the previous studies which our study replicates, utilised the empathic sadness scale of the IECA because this was found to be a specific factor within the Dutch version (De Wied et al. 2007b). This does however limit results to one emotion within affective empathy. Empathic sadness is measured in the IECA via questions about one’s response to observing another crying, which, as we previously pointed out, may be seen as a more feminine than masculine expression (Van Tilburg et al. 2002). Other emotions within affective empathy such as empathic anger or empathic pain may show different gender patterns because their expression may hold different nuances as to whether they are considered feminine or masculine or neither. Future research could investigate whether there are gender differences in same-sex vs other-sex affective empathy in adolescents when other emotions are considered.

Fourth, we cannot ignore that the two samples, due to streaming, represent different education levels in the Netherlands, which may limit our findings’ generalisability. Future studies would benefit from recruiting a sample where education level is evenly distributed to rule out any impact of education level. However, our study has demonstrated gender differences in same-sex and other-sex empathic sadness within two education levels, which could be seen as a strength. Finally, future studies would benefit from recruiting more waves of information covering the complete age range of adolescence in order to make more robust statements regarding the development of affective empathy over adolescence.

### Practice Implications

Studying empathy is important given its functions in maintaining and promoting relationships on an individual and societal level. The results of our study indicate patterns on the group level and give an insight into the nuances of empathic sadness in adolescents furthering our understanding of what empathic abilities they are expressing at this stage. What cannot be deduced from our study is whether empathy plays a similar role in social bonding across all same-sex and other-sex adolescent relationships: within friendships, romantic relationships or relationships with strangers. This limitation is because our study assessed empathic sadness towards male and female targets *in general* through the “boy” and “girl” items of the IECA. Also we cannot speculate about what the expression of empathic sadness across different relationships may mean for these relationships in adolescents. However, these remain interesting questions, and answers would inform our understanding of the role of empathy within adolescent relationships.

Research has shown that within male adolescents’ same-sex friendships, young men avoid expressing emotion and pain, and they discourage others from doing the same or sharing emotions (Oransky and Marecek 2009), as well as lack emotional intimacy and trust (Way 1997)—qualities reminiscent of low affective empathy. However, this is not to say that male same-sex friendships are not experienced as being close. McNelles and Connolly (1999) found that although male and female adolescents did not differ in their experience of intimacy in same-sex friendships, female adolescent were more likely to establish intimacy through discussion and self-disclosure whereas male adolescents were more likely to do this through shared activities. Such research indicates that adolescents’ same-sex friendships have different characteristics between genders and that these characteristics still allow close, intimate relationships. To take our results further and to answer such questions, future research would be needed on the role of affective empathy in same-sex and other-sex relationships of different qualities in adolescence and what the influence of patterns, as found here, may have on theses relationships.

Empathy can assist in inhibiting unwanted adolescent behaviours such as aggression (Jolliffe and Farrington 2004). Yet during adolescence, social conflict occurs between the adolescent with parents, peers, and sometimes society (e.g., through delinquency). Knowing that male adolescents show less empathic sadness toward others, particularly male targets, than do female adolescents is helpful when assisting adolescents to resolve conflict or understand what has gone wrong in an interaction. Rather than suggesting that male adolescents consider how they have made the other person feel, perhaps other skills could be drawn on, for example, perspective taking. Perspective taking is more cognitive and more akin to the processing done by males (Derntl et al. 2010) and may be more helpful to male adolescents in the light of the current findings when resolving or understanding social conflicts, particularly with other male targets. Asking female adolescents to consider how they have made another person feel may be more productive given, on average, they show more empathic sadness than do male adolescents. Investigating affective empathy in adolescents is important to allow those working with this age group to understand better the nuances of social cognitive processes occurring in order to help them navigate and understand the social world, particularly that of adults.

Current findings may be used to improve understanding of the effectiveness of empathy training programs; for example, empathy training may show lower effectiveness in enhancing empathy of male adolescents towards male targets, particularly when compared to the effectiveness in enhancing their empathy towards female targets. Such training may benefit from an understanding of how adolescents respond to others of the same and other-sex, and how this develops over time. Empathy training has been recommended by research, and used, as part of an intervention for adolescent bullying (Ang and Goh 2010) and aggressive behaviours (Björkqvist et al. 2000).

### Conclusions

Our study establishes gender differences in same-sex versus other-sex affective empathy in Dutch samples, both cross-sectionally and longitudinally. The cross-sectional data confirm gender differences in other-sex and same-sex empathy, and the longitudinal data suggests that these differences remain stable over time. Results are in line with an evolutionary perspective and consideration of the biological and social changes occurring in adolescence. Our study suggests differentiating between other-sex and same-sex empathic sadness may prove a useful distinction when considering adolescents’ empathic abilities and related interventions. Future research should investigate other emotions within affective empathy and consider potential mechanisms through which these gender differences may occur (for example, competition and co-operation). Overall, our findings provide insight into the nuances of adolescents’ affective empathy development and highlight the importance of considering same-sex versus other-sex empathy in research on adolescents’ empathy.
